# Psychedelika und Dissoziativa in der Psychiatrie: Herausforderungen in der Behandlung

**DOI:** 10.1007/s00115-024-01727-0

**Published:** 2024-08-28

**Authors:** Johannes Jungwirth, Francesco Bavato, Boris B. Quednow

**Affiliations:** 1 AG Neurophänomenologie des Bewusstseins, Erwachsenenpsychiatrie und Psychotherapie, Psychiatrische Universitätsklinik Zürich, Universität Zürich, Zürich, Schweiz; 2 AG Experimentelle Pharmakopsychologie und psychologische Suchtforschung, Erwachsenenpsychiatrie und Psychotherapie, Psychiatrische Universitätsklinik Zürich, Universität Zürich, Zürich, Schweiz

**Keywords:** Ketamin, Behandlungsrisiken, Nebenwirkungen, Abhängigkeitspotenzial, Studienqualität, Ketamine, Treatment risks, Side effects, Addictive potential, Study quality

## Abstract

Mit der Entdeckung der antidepressiven Effekte des Ketamins und dem zunehmenden Rückzug der Pharmaindustrie aus der Entwicklung neuer Psychopharmaka ist die psychiatrische Forschung zur klinischen Anwendung von Halluzinogenen in der Psychiatrie in den letzten zwei Dekaden geradezu erblüht. Vielversprechende Befunde zu verschiedenen Therapieansätzen mit Psychedelika, wie Lysergsäurediethylamid (LSD) und Psilocybin, und Dissoziativa, wie Ketamin und Esketamin, haben in den letzten Jahren bei Forscher:innen, Kliniker:innen und Patient:innen große Hoffnungen geweckt, sodass bereits von einer Zeitenwende in der Psychiatrie die Rede war. Als eine der ersten dieser Substanzen wurde intranasales Esketamin im Dezember 2019 in den USA und der EU für die Behandlung der therapieresistenten Depressionen zugelassen, die Schweiz zog 2020 nach. Psilocybin kann seit kurzem in Australien, Kanada und der Schweiz in Ausnahmefällen für die Depressionsbehandlung eingesetzt werden, während derzeit große Zulassungsstudien mit verschiedenen Psychedelika weltweit auf dem Weg sind. Psychedelika und Ketamin/Esketamin gelten in der medizinischen Anwendung als sicher. Doch wie bei jeder neuen Therapie ist es von entscheidender Bedeutung, neben den Hoffnungen auch die spezifischen Herausforderungen dieser neuen Therapieansätze sorgfältig zu betrachten und zu beurteilen. Überhöhte Erwartungen und eine unzureichende Risiko-Nutzen-Abschätzung schaden den Patient:innen und dem Ansehen der Behandelnden. Während bereits von einem möglichen Paradigmenwechsel in der psychischen Gesundheitsversorgung gesprochen wird, soll in dieser Überblicksarbeit der Fokus bewusst auf die möglichen Risiken der Behandlung und die methodischen Schwächen bisheriger Studien gerichtet werden.

## Abstract (en)

With the discovery of the antidepressive effects of ketamine and the increasing withdrawal of the pharmaceutical industry from the development of new psychotropic drugs, the psychiatric research into the clinical application of hallucinogens in psychiatry has literally blossomed in the last two decades. Promising results for various treatment approaches with psychedelic agents, such lysergic acid diethylamide (LSD) and psilocybin, and dissociative agents, such as ketamine and esketamine, have raised great hopes among researchers, clinicians and patients in recent years, so that there was already talk of a new era in psychiatry. As one of the first of these substances, in December 2019 intranasal esketamine was approved in the USA and the EU for the treatment of treatment-resistant depression and Switzerland followed in 2020. Recently, psilocybin was approved in Australia, Canada and Switzerland for compassionate use in exceptional cases for the treatment of depression, while large approval studies with various psychedelic agents are currently ongoing worldwide. The medical application of psychedelic agents and ketamine/esketamine is considered to be safe; however, as with all new forms of treatment it is of crucial importance that, in addition to the hopes, the specific challenges of these new treatment approaches must also be carefully considered and assessed. Excessive expectations and an insufficient risk-benefit estimation are detrimental to the patients and the reputation of the treating physician. Although a possible paradigm shift in the care of mental health is already being discussed, this review article consciously concentrates on the possible risks of treatment and the methodological weaknesses of the studies carried out so far.

## Hintergrund

Klassische *Psychedelika* sind halluzinogene Substanzen, die durch Stimulation bestimmter Serotoninrezeptoren (vor allem über 5‑HT_2A_) zu einer dosisabhängigen Veränderung der Wahrnehmung, des Bewusstseins und der Emotionsverarbeitung führen [53]. Die wichtigsten Vertreter, die derzeit hinsichtlich ihres Nutzens in der Psychiatrie beforscht werden, sind Psilocybin, Lysergsäurediethylamid (LSD) und Dimethyltryptamin (DMT, Hauptbestandteil des „Ayahuasca“). Erste Metaanalysen ergaben signifikante und schnelle Symptomverbesserungen bei depressiven Erkrankungen und Angststörungen nach Verabreichung von Psilocybin, Ayahuasca oder LSD in Kombination mit Psychotherapie [35, 38, 58].

Eine kürzlich erschienene Übersicht, welche 59 Studien zur Therapie mit Psychedelika inkludierte, zeigte große Effektstärken bei zum Teil schwer zu behandelnden psychiatrischen Erkrankungen wie der (therapieresistenten) Depression, Angst und Depression bei terminaler Krankheit, Substanzgebrauchsstörungen, Zwangsstörungen, Anorexia nervosa und andere. Unter den klassischen Psychedelika zeigte Psilocybin (Hedges’ g = 1,49) die stärkste therapeutische Wirkung gefolgt von DMT (Hedges’ g = 1,34) und LSD (Hedges’ g = 0,65; [78]). Auch die Langzeiteffekte von Psilocybin mit begleitender Psychotherapie erscheinen vielsprechend [29]. Eine neuere Übersichtsarbeit, die 44 Studien umfasste, stufte klassische Psychedelika zudem als sicher und gut verträglich ein [8].

Phencyclidin und Ketamin wurden ursprünglich als Anästhetika entwickelt

*Dissoziativa*, wie Phencyclidin (PCP, „Angel Dust“), Ketamin und sein S(+)-Enantiomer Esketamin, stellen neben den Psychedelika und Delirantia (z. B. Muscimol, Scopolamin) eine weitere Untergruppe von Halluzinogenen dar. PCP und Ketamin wurden ursprünglich als Anästhetika entwickelt. Da diese Substanzen analgetisch und kataplektisch wirken, aber nur eine oberflächliche Narkose bewirken, wurden sie auch als dissoziative Anästhetika bezeichnet. Es wird davon ausgegangen, dass Ketamin und Esketamin durch die Blockade des glutamatergen N‑Methyl-D-Aspartat(NMDA)-Rezeptors im Gehirn ihre psychoaktiven Wirkungen entfalten. Dieser Rezeptortyp spielt eine wesentliche Rolle in der Regulierung synaptischer Plastizität und ist in viele Lern- und Gedächtnisfunktionen eingebunden. Ketamin verstärkt zudem die Dopaminausschüttung und aktiviert, wenn auch schwach, μ‑Opioid-Rezeptoren [41, 49].

Eine systematische Untersuchung der Effektivität von Ketamin bei therapieresistenter Depression zeigte über 79 Studien hinweg, dass Ketamin kurzfristig effektiv wirksam ist und dass der Effekt bei wiederholten Behandlungen auch anhalten kann [2]. Darüber hinaus scheint Ketamin auch zur Suizidprävention geeignet zu sein [43]. Jüngste Daten zur Sicherheit intranasalen Esketamins bei affektiven Störungen deuten auf ein günstiges Nebenwirkungsprofil hin [54].

## Herausforderungen in der Psychedelikabehandlung

Eine grundsätzliche Herausforderung in der Beurteilung des Risiko-Nutzen-Verhältnisses von Psychedelika besteht in der Definition eines unerwünschten Ereignisses. Die Unterscheidung zwischen therapeutisch gewünschter Wirkung und potenziell schädlicher Nebenwirkung ist im psychedelischen Kontext nicht immer eindeutig [64]. Auch erfolgte bislang die Erfassung unerwünschter Ereignisse oft lückenhaft und uneinheitlich, sodass das Sicherheitsprofil der Psychedelika derzeit nur unvollständig kartiert ist [8, 64].

### Akute Nebenwirkungen

Die am häufigsten berichteten akuten Nebenwirkungen nach Psilocybingabe sind Kopfschmerzen (24 %), Übelkeit (22 %) sowie Schwindel und Müdigkeit (jeweils 6 %; [26]). Diese Nebenwirkungen sind in aller Regel leicht bis moderat sowie vorübergehend. In seltenen Fällen können schwerwiegende Nebenwirkungen wie psychotische oder traumatische Erfahrungen (sog. „Bad Trips“) auftreten. Solche intensiven emotionalen Erlebnisse, die mit Angst, Dysphorie und Paranoia einhergehen können, sind in der Regel von kurzer Dauer, könnten aber zu einer vorübergehenden Labilisierung, zu einer Verschlimmerung der Symptome oder einer Retraumatisierung beitragen [22]. Ob „Bad Trips“ auch wertvolle Erfahrungen für den Behandlungsprozess sein können, wird noch diskutiert [25].

Wechselwirkungen mit anderen serotonergen Substanzen können weitere Risiken bergen, jedoch gilt bspw. das Auftreten eines vollen Serotoninsyndroms in Kombination mit serotonergen Antidepressiva als selten oder benigne [42]. Die Kombination von Psychedelika mit Monoaminooxidasehemmern ist wegen eines stark erhöhten Risikos für ein Serotoninsyndroms, mit Lithium wegen eines erhöhten Risikos für einen epileptischen Anfall jedoch kontraindiziert [42].

### Subakute und persistierende Nebenwirkungen

Zu den möglichen schweren Nebenwirkungen, die auch nach einer psychedelischen Behandlung auftreten können, gehören psychotische Episoden, halluzinogenbedingte anhaltende Wahrnehmungsstörungen („hallucinogen persisting perception disorder“ [HPPD], auch „Flashbacks“ genannt), klinische Exazerbationen und Suizidalität.

Die Entstehung einer chronischen Psychose nach Verabreichung von Psychedelika ist selten und wird aufgrund strengerer Ausschlusskriterien in klinischen Kontexten kaum beobachtet [79]. Menschen mit genetischer Prädisposition für psychotische oder bipolare Erkrankungen wurden jedoch bislang auch von der Behandlung und klinischen Forschung meist ausgeschlossen [24]. Bei frühen Studien mit LSD zeigte sich bei 1200 Teilnehmern [15] bzw. 247 Teilnehmern [45] jeweils ein einzelner Fall einer psychotischen Reaktion, die länger als 48 h anhielt. Die Prävalenz für die Entwicklung einer Psychose nach einer experimentellen LSD-Verabreichung wird in der Literatur mit einer Spanne von 0,08–4,6 % angegeben, wobei psychiatrische Patient:innen besonders häufig betroffen waren [20]. Im Rahmen moderner Psychedelikastudien scheinen solche Fälle aber seltener beobachtet worden zu sein [79], wahrscheinlich bedingt durch die strengeren, aber möglicherweise nicht immer realistischen Ein- und Ausschlusskriterien.

Treten Wochen oder Monate nach Substanzgabe wiederholt und unerwartet halluzinogenartige Wahrnehmungsveränderungen auf, spricht man von einer HPPD („Flashbacks“). Nach der Verabreichung von LSD und Psilocybin berichteten 13 der 142 gesunden Proband:innen (9,2 %) von wiederkehrenden Flashbacks, wobei in keinem dieser Fälle die Kriterien einer HPPD erfüllt waren und die Phänomene primär als mild wahrgenommen und daher letztlich als nicht klinisch relevant eingeschätzt wurden [51]. Laut einer großen Onlineumfrage erleben jedoch etwa 60 % der LSD-Konsumenten HPPDs und 4 % der Konsumenten berichten von HPPDs, die als psychisch belastend empfunden werden [3]. Insbesondere psychologische Langzeiteffekte werden derzeit in der Erfassung vernachlässigt [9]. Psychologische Risiken, wie die Verschlechterung der Stimmung und der Verlust emotionaler Stabilität, sind wenig untersucht und könnten gravierender sein als bisher angenommen ([9]; siehe auch Fallbeispiel unten). Zudem ist, wie oben beschrieben, die Bewertung psychologischer Effekte, wie die Selbstentgrenzung, die Veränderung der Lebensperspektive und Aufweichung von Abwehrmechanismen, schwierig und bedarf weiterer Untersuchungen [64]. Eine Metaanalyse über drei Studien zeigte jedoch keine stärkere Symptomverschlechterung nach einer Psychedelikabehandlung als in der Standardbehandlung mit Escitalopram (je ca. 10 %; [67]).

Wichtig ist das „Set und Setting“, d. h. die mentale Verfassung des Betroffenen und die Umgebung

Es wurde kürzlich im Rahmen einer klinischen Studie berichtet, dass in den Psilocybinbedingungen Suizidideen und selbstverletzenderes Verhalten häufiger auftraten als in der Placebobedingung [26]. In ihrer systematischen Übersichtsarbeit, welche den Zusammenhang zwischen klassischen Psychedelika und Suizidalität untersuchte [80], wurde jedoch postuliert, dass eine psychedelische Therapie die Suizidalität in bestimmten psychiatrischen Populationen auch verringern könnte. In unsicheren und nicht überwachten Umgebungen scheint eine Therapie mit Psychedelika aber auch suizidales Verhalten auslösen zu können [36]. Epidemiologische Studien, die einen Zusammenhang zwischen dem nichtmedizinischen Konsum von Psychedelika und Suizidalität untersuchten, berichteten widersprüchliche Befunde [65] oder eher positive Effekte auf die Suizidalität [31]. Diese Beobachtungen weisen auf die Wichtigkeit von „Set und Setting“ (d. h. die mentale Verfassung des Betroffenen und die Umgebung) im Hinblick auf den Sicherheitsaspekt sowie auf die Einbettung in einen interdisziplinären Therapieprozess hin.

Akut beeinträchtigen Psychedelika die Aufmerksamkeit, Gedächtnis und exekutive Funktionen [4, 60]. Die möglichen langfristigen Auswirkungen von Psychedelika auf die kognitiven Funktionen sind noch nicht geklärt [63], obwohl die Substanzen akut und postakut starke neuroplastische Effekte auf das Serotoninsystem ausüben können [74]. Ein regelmäßiger Konsum psychedelischer Substanzen wurde bereits mit strukturellen Veränderungen in den Hirnbereichen assoziiert, die mit Aufmerksamkeitsprozessen und selbstreferenziellen Denkprozessen assoziiert sind [7]. Diese Veränderungen könnten berichteten Persönlichkeitsveränderungen bei Langzeitkonsumenten zugrunde liegen [7]. Dass Neuroplastizität auch in Neurotoxizität übergehen kann, wurde zumindest für hochaffine 5‑HT_2A_-Rezeptor-Agonisten wie die Psychedelika 2,5-Dimethoxy-4-iodamphetamin (DOI; [12, 13]) und 5‑Methoxy‑N,N‑diisopropyltryptamin (5-MeO-DIPT, Slang: „Foxy“; [56]) im Tierversuch demonstriert, da eine sehr starke oder anhaltende Aktivierung des 5‑HT_2A_-Rezeptors die Apoptose von Neuronen auslösen kann [62]. Dies spricht möglicherweis dafür, dass für die klinische Anwendung eher Psychedelika mit moderater Affinität am 5‑HT_2A_-Rezeptor vorbehalten sein sollten. Tiefgreifende Untersuchungen über die Auswirkungen klassischer Psychedelika auf die mikrostrukturelle Hirnintegrität beim Menschen stehen jedoch noch aus.

### Abhängigkeitspotenzial

In der suchtmedizinischen Praxis spielen serotonerge Psychedelika aufgrund ihres geringen Abhängigkeitspotenzials kaum eine Rolle [65]. Psychedelika sind aber in der Partyszene sehr verbreitet und ihr Konsum ist mit einer höheren Wahrscheinlichkeit für Risikoverhaltensweisen und nachteilige gesundheitlichen Folgen verbunden [23].

### Studienqualität

Die Qualität der klinischen Psychedelikastudien wird bisher durch verschiedene methodische Herausforderungen begrenzt. Ein grundlegendes Problem ist das Fehlen von Gruppenvergleichen mit aktivem Placebo [11]. Die einzigartigen und sofort wahrnehmbaren psychoaktiven Effekte der Psychedelika lassen sich sowohl durch Studienteilnehmende als auch Forschende leicht erkennen (sog. „funktionelle Entblindung“). Die Benennung dieser Studien als „doppelblind, placebokontrolliert“ muss daher hinterfragt werden [10].

Die hohen Erwartungen an psychedelische Substanzen (u. a. ausgelöst durch weltweite affirmative Berichterstattung), besonders gepaart mit einer erhöhten Suggestibilität durch die Substanzen [14], kann bei den Studienteilnehmenden zu starken Placeboeffekten führen [30]. In den Placebogruppen kann dies wiederum durch Enttäuschung zu ausgeprägten negativen Effekten (Noceboeffekt) führen. Die Nutzung dieser Suggestibilität zur Förderung therapeutischer Placeboeffekte („Super-Placebo“) wurde vorgeschlagen, hat jedoch ethische und wissenschaftliche Grenzen [19]. Interessanterweise zeigen die Kurven der meisten veröffentlichten Studien, im Gegensatz zu Studien mit konventionellen Antidepressiva, kaum Verbesserung in der Placebogruppe, was die Bedeutung des Noceboeffektes in diesen Gruppen illustriert (vergleiche [76]).

In den bisherigen klinischen Studien wurden weder die funktionelle Entblindung noch die Erwartungshaltung jedoch ausreichend berücksichtigt [69].

Viele Psychedelikaforscher weisen zudem eine starke Identifikation mit diesem therapeutischen Ansatz auf [34], was zu einer hohen Behandlungsmotivation führt und auch bei der Entwicklung von Placebo- und Noceboeffekten eine Rolle spielen könnte. Dadurch besteht das Risiko einer Bestätigungsverzerrung („confirmation bias“ oder „experimenter bias“), da die hohen Erwartungen und die große Motivation der Forscher dazu führen können, die Interpretation der Daten bewusst oder unbewusst zu beeinflussen. Darüber hinaus kann es zu einer Selektionsverzerrung kommen, da es sich bei den Studienteilnehmern um therapiemotivierte Personen handelt, die eine positive oder zumindest nicht negative Einstellung zum Konsum von Psychedelika haben (siehe auch [48]).

Diese methodischen Mängel müssten behoben werden, um die Validität und Zuverlässigkeit dieser Forschung zu verbessern und fundierte klinische Empfehlungen abzuleiten.

### Ethik

Ethische Herausforderungen der Therapie betreffen insbesondere die individuelle Patient:innenautonomie und gesundheitspolitische Implikationen. Ein Aspekt betrifft die Frage der Einwilligung in die Behandlung („informed consent“; [44]). Die tiefgreifende, oft als spirituell oder transformativ beschriebene Erfahrung, die durch Psychedelika hervorgerufen wird, führt zu der Frage, inwieweit Patient:innen vollständig verstehen und antizipieren können, worauf sie sich einlassen. Die Fähigkeit, eine informierte Zustimmung zu geben, setzt voraus, dass Patient:innen die potenziellen Risiken und Nebenwirkungen verstehen. Doch bei Therapieformen, die bewusstseinsverändernde Erfahrungen induzieren, wird dieses Verständnis herausgefordert. Wie können wir sicherstellen, dass Patient:innen wirklich verstehen, was eine psychedelische Erfahrung beinhaltet und was sich daraus ergibt [44]?

Für die negative Instrumentalisierung von Psychedelika gibt es historisch und zeitgenössisch zahllose Beispiele

Psychedelika können die Suggestibilität erhöhen [14] und psychologische Abwehrmechanismen reduzieren [72]. Dies stellt ein potenzielles Risiko dar, da die mediale Aufmerksamkeit, die Idealisierung dieser Substanzen und die geringe Verfügbarkeit von Behandlungsplätzen ein Umfeld schaffen können, in dem die Überhöhung von Therapeut:innen, Elitismus und letztlich Guruismus gedeihen können. Für die negative Instrumentalisierung von Psychedelika in Sekten und bei selbsternannten Gurus gibt es historisch und zeitgenössisch zahllose Beispiele [17, 18]. Hier sind strenge ethische Richtlinien und eine sorgfältige Überwachung erforderlich, um einen potenziellen therapeutischen Machtmissbrauch zu verhindern. Die Erwartung, dass psychedelisch unterstützte Psychotherapie neue Bedeutungen (z. B. neue Selbsterzählungen) schaffen soll, erhöht zudem die Möglichkeit, dass die Therapeut:innen eigene Lebensvorstellungen in die behandelte Person projizieren, insbesondere in einem Kontext erhöhter Suggestibilität [52].

Die Diskussion darüber, ob Psychedelika eigenständig wirken oder von der Wirkung begleitender Psychotherapie abhängig sind, beeinflusst zudem den Regulierungsprozess und stellt eine Herausforderung bei der Standardisierung von Therapieprogrammen dar [27, 28].

### Fallbeispiel 1

Ein 52-jähriger Patient mit jahrzehntelanger rezidivierender depressiver Störung nach ICD-10 (International Statistical Classification of Diseases and Related Health Problems 10) wurde mit einer mittelgradigen Episode vorstellig (Montgomery-Åsberg Depression Rating Scale [MADRS] 21 Punkte, Beck-Depressions-Inventar [BDI] 17 Punkte). Trotz zahlreicher vorheriger Behandlungen, einschließlich selektiver Serotonin-Wiederaufnahmeinhibitoren (SSRIs), Serotonin-Noradrenalin-Wiederaufnahmeinhibitoren (SNRIs) sowie Augmentation mit Lithium, Quetiapin und Esketamin, zeigte er keine ausreichende Verbesserung. Psychotherapeutische Ansätze hatten ebenfalls keine bedeutsame Linderung gebracht. Eine Diagnostik mittels SKID-2-Interview (Strukturiertes Klinisches Interview für Diagnostic and Statistical Manual of Mental Disorders IV) zeigte keine Persönlichkeitsstörung.

Nach Erhalt einer Ausnahmebewilligung durch das schweizerische Bundesamt für Gesundheit wurde eine Behandlung mit Psilocybin initiiert, beginnend mit fünf vorbereitenden Psychotherapiestunden, in denen unter anderem biografische Konflikte, Erwartungen und die Zielsetzung der Behandlung thematisiert wurden. Während der ersten Behandlungssitzung mit 20 mg Psilocybin erlebte der Patient traumartige Visionen und Gefühle der Trauer, die in den Nachbesprechungen als Teil eines Ablöseprozesses besprochen wurden.

Wenige Tage nach der Behandlung verschlechterten sich jedoch seine Symptome signifikant, mit einer Zunahme von innerer Unruhe, Anspannung und Angst (MADRS 26, BDI 26). Der Patient beschrieb sich selbst als „hoffnungslosen Fall“. Es erfolgten psychotherapeutische Sitzungen über einen Zeitraum von 3 Monaten ohne eine Verbesserung. Auf Wunsch des Patient:innen wurde schließlich eine zweite Sitzung mit erhöhter Dosis (30 mg) durchgeführt, in der der Patient tiefgreifende Dankbarkeit und Verbundenheit empfand. Außerdem beschrieb er Erkenntnisse, einem „äußeren Ideal nicht hinterherrennen“ zu müssen. Er verließ unsere Klinik in deutlich gebessertem Zustand (MADRS 10, BDI 9). Diese positive Wende hielt jedoch nicht an – nach einer familiären Belastungsprobe verschlechterten sich seine Symptome kurz darauf erneut (MADRS 25, BDI 23). Nach einer Stabilisierungsphase mit leichter Verbesserung (MADRS 16, BDI 21) wurde mit dem Patient:innen nach weiteren 4 Monaten eine dritte Psilocybinbehandlung geplant, um den positiven Prozess wieder aufzunehmen.

Die dritte Behandlungssitzung mit 35 mg führte zu einer tiefen emotionalen Auseinandersetzung mit einem bisher unbekannten Trennungsschmerz von seiner Mutter, welche die Familie verlassen hatte, als der Patient ein Kind war. Eine anschließende Besserung der Symptomatik hielt wieder nur wenige Tage an, sodass er einen Monat nach der dritten Behandlung insgesamt von einer Verschlechterung berichtete. Jede Kleinigkeit sei ihm zu viel, er sei sehr enttäuscht von sich und seinem Verlauf. Nichts helfe ihm, er wisse nicht, was er ändern solle. Er habe keine Energie, wolle nicht aus dem Haus gehen, fühle sich nutzlos. Die letzte Psilocybinsitzung sitze ihm „noch in den Knochen“ und ähnlich klingende Lieder wie die, die während der Psilocybinbehandlung abgespielt wurden, würden ihn erschrecken und traurig machen (MADRS 25, BDI 22).

Dieses Beispiel unterstreicht die Komplexität der Therapie mit Psychedelika. Es zeigt, dass trotz tiefgreifender Einsichten und vorübergehender Verbesserungen die langfristige Stabilität der Symptomlinderung nicht garantiert ist und in manchen Fällen eine Verschlechterung nach der Behandlung eintreten kann.

## Herausforderungen in der Ketaminbehandlung

Eine besondere Herausforderung bei der klinischen Anwendung von Ketamin besteht in der Definition der Dauer der Behandlung. Während die meisten Studien die kurzfristige Sicherheit und Verträglichkeit von Ketamin nach wenigen Anwendungen bewerteten, legen die aktuellen klinischen Befunde die Notwendigkeit einer längeren Behandlung nahe [68]. Für die langfristige Sicherheit einer verlängerten Ketaminbehandlung im psychiatrischen Kontext liegen erste positive Erkenntnisse vor [46]. Indirekte Hinweise auf potenzielle Nebenwirkungen und neurobiologische Folgen eines chronischen Ketamineinsatzes in anderen klinischen Kontexten (z. B. in der Schmerztherapie) und im Bereich des nichtmedizinischen Gebrauchs sollten aber berücksichtigt werden.

### Akute Nebenwirkungen

Akute Nebenwirkungen im Zusammenhang mit der einmaligen Verabreichung von Ketamin sind häufig, wobei sie meistens vorübergehender Natur sind und spontan abklingen. In einer systematischen Überprüfung von 60 klinischen Studien zur Behandlung von Depressionen mit Ketamin waren die häufigsten akuten psychiatrischen Nebenwirkungen dissoziative Zustände, Sedierung, Angst, Unruhe, Reizbarkeit, Euphorie, Wahnvorstellungen, formale Denkstörungen, Panik und Apathie [66]. Im Allgemeinen berichteten Studien, die den intravenösen Verabreichungsweg nutzten, über mehr psychotomimetische oder dissoziative Nebenwirkungen als Studien, die andere Verabreichungswege nutzten (z. B. oral, subkutan, intramuskulär; [66]).

Zu den häufigsten akuten somatischen Nebenwirkungen von Ketamin gehören Kopfschmerzen, Schwindel, Bluthochdruck, Tachykardie, Herzklopfen, Herzrhythmusstörungen, Brustschmerzen, Schwindel, aber auch Blutdruckabfall und verminderte Herzfrequenz. Im Allgemeinen klangen diese Wirkungen innerhalb von 90 min nach der verabreichten Dosis ab [66]. Höhere Dosen (über den medizinischen Gebrauch hinaus) können zu Nystagmus, Ataxie, Dysarthrie, Muskelsteifheit bis hin zu Krampfanfällen und Koma führen [49].

Nach akuter Gabe beeinträchtigen sowohl intravenöses Ketamin als auch intranasales Esketamin verschiedene kognitive Funktionen [16, 50]. Diese Defizite gehen nach intravenöser Gabe von Ketamin erst nach etwa einem Tag [16], bei intranasaler Gabe bereits nach 2 h wieder auf das Placeboniveau zurück [50].

### Subakute und persistierende Nebenwirkungen

Zu den häufigsten verzögerten psychischen Nebenwirkungen des Ketamins gehören Angstzustände, Verschlechterung der Depression, Suizidalität und Hypomanie, wobei unklar ist, ob diese tatsächlich mit der Ketaminbehandlung in Zusammenhang stehen [66]. Die Sicherheit einer langfristigen, wiederholten Ketaminverabreichung, wie sie in der klinischen Praxis zunehmend praktiziert wird, ist bislang aber noch kaum untersucht worden.

Im Rahmen eines andauernden nichtmedizinischen Konsums weist Ketamin psychoseinduzierende Effekte auf, die mit chronischen Veränderungen im Glutamatsystem erklärt werden [75, 77]. Nach kurzzeitiger experimenteller Verabreichung bei mehr als 2000 gesunden Proband:innen konnte hingegen keine anhaltende Psychose beobachtet werden [59]. Es fehlt aber bisher an systematischen Untersuchungen hinsichtlich des Psychoserisikos im Rahmen einer länger andauernden Ketaminbehandlung. Das Risiko einer Verschlimmerung einer bereits bestehenden psychotischen Erkrankung ist bei der Einnahme von Ketamin erhöht; daher müssen psychotische Störungen zu Beginn der Behandlung abgeklärt werden [37].

Chronischer nichtmedizinischer Gebrauch kann zu strukturellen Veränderungen im Gehirn führen

Bei chronischem nichtmedizinischem Gebrauch kann Ketamin neurotoxisch wirken und zu strukturellen und funktionellen Veränderungen im Gehirn führen [39, 75], was sich auch in Form kognitiver Defizite, insbesondere im Arbeits- und Langzeitgedächtnis, äußern kann [49]. Ob eine medizinische Langzeitbehandlung die kognitive Leistungsfähigkeit beeinflussen kann, muss noch geprüft werden.

Ketamin und seine Metaboliten können leber-, nieren- und blasentoxisch wirken, was zu Unterleibsschmerzen, Dysurie und Hämaturie führen kann [40]. Diagnostisch findet sich bei nichtmedizinischen Konsumenten oft ein verkleinertes Blasenvolumen, eine Verdickung der Blasenwand, eine Dilatation des Harnleiters und perivesikale Entzündungen. Potenziell schwerwiegende und möglicherweise anhaltende toxische Wirkungen auf das Leber- und Nierensystem wurden aber auch bei der wiederholten medizinischen Verabreichung von Ketamin zur Behandlung chronischer Schmerzen gezeigt [5, 40, 55]. Bei Patient:innen, die Esketamin/Ketamin in einer Dosierung erhalten, die den Verschreibungsrichtlinien für Depressionen entspricht, wurden bisher keine schweren Blasen- und Lebererkrankung festgestellt, doch auch diese können leichtere urologische Beschwerden hervorrufen [54].

### Abhängigkeitspotenzial

Regelmäßiger nichtmedizinischer Konsum kann in eine Ketamingebrauchsstörung münden, die durch Toleranzentwicklung und ein psychologisches Entzugssyndrom („craving“) gekennzeichnet ist, jedoch nur mit einer begrenzten körperlichen Abhängigkeit oder Entzugssymptomatik einhergeht [33, 49]. Experten schätzen, dass Ketamin ein ähnliches Abhängigkeitspotenzial aufweist wie Cannabis und Alkohol [57]. In Südostasien ist Ketamin eine der am häufigsten gebrauchten Substanzen unter jungen Erwachsenen und Ketaminkonsumstörungen treten in dieser Region viel häufiger auf als in Europa [32]. Der wahrscheinliche Rückgang der Stigmatisierung des Ketaminkonsums durch die medizinische Anwendung [71], der Anstieg der Prävalenz des Freizeitkonsums [21] und sogar Berichte über eine derzeitige Ketamin-Mode („Ketamin-Chic“, [21]) legen nahe, den weiteren Verlauf des nichtmedizinischen Konsums auch in Europa gut im Blick zu behalten.

Das Risiko einer Ketamingebrauchsstörung im Rahmen einer psychiatrischen Behandlung wurde bisher kaum untersucht, aber Fälle von Abhängigkeit durch Selbstmedikation von Depressionen sind bekannt [6]. Es wird daher empfohlen, Ketaminbehandlungen im Hinblick auf den Risiko-Nutzen-Aspekt ähnlich wie medizinische Stimulanzien oder Benzodiazepine zu betrachten [70].

## Diskussion

Psychedelika und Ketamin zeigen ein gutes Wirksamkeits- und Sicherheitsprofil [54, 68, 78]. Damit stellen sie eine vielsprechende potenzielle Behandlungsoption für verschiedene psychiatrische Indikationen dar. Obwohl die vorhandenen Studienergebnisse ermutigend sind, dürfen die damit verbundenen Herausforderungen und Risiken nicht unterschätzt werden. Die zunehmende Aufmerksamkeit und die Erwartung auf baldige Zulassungsprozesse bedingen die Notwendigkeit einer differenzierten Betrachtung ihrer Wirkmechanismen, Wirksamkeit und potenziellen Risiken. Die Hoffnung auf eine baldige Zulassung von Psychedelika wurden aber jüngst durch die Ablehnung von 3,4-Methylendioxy-N-methylamphetamin (MDMA) zur Behandlung der Postraumatischen Belastungsstörung durch die amerikanische Zulassungebehörde FDA getrübt [61].

Studien mit aktiven Placebos und Cross-over-Designs würden zu einer valideren Evidenzlage beitragen

Die somatischen Risiken scheinen für beide Substanzklassen bei therapeutischer Gabe gering zu sein. Bei langfristigem nichtmedizinischem Ketaminkonsum, aber auch im Rahmen einer Ketaminbehandlung im Schmerzkontext sind jedoch relevante Nebenwirkungen auf die Leber- und Blasenfunktion bekannt. Die langfristigen psychologischen und neuropsychiatrischen Effekte sind bei Psychedelika noch nicht ausreichend erforscht. Während kurzfristige Untersuchungen positive Ergebnisse zeigen, bedarf es langfristiger und spezifischer Beobachtungen, um dauerhafte neurologische Schäden oder Verschlechterungen der psychischen Gesundheit zu bewerten. Die individuelle Anpassung der Therapie an den Patient:innen, das „Set und Setting“ und die professionelle Begleitung durch geschultes Personal scheinen entscheidend zu sein, um die Sicherheit und Effektivität der Behandlung zu gewährleisten. Die Integration von Psychedelika in die bestehenden therapeutischen Rahmenwerke und die damit verbundene Standardisierung von Behandlungsprotokollen ist daher eine besondere Herausforderung für die Zukunft. Auch die Rolle der Psychotherapie an den Behandlungseffekten ist bislang nicht ausreichend geklärt und muss Gegenstand weiterer Forschung sein [1].

Ein kritischer Blick auf die Studienqualität offenbart, dass die Identifizierbarkeit von Verum und Placebo (funktionelle Entblindung) die Ergebnisse verzerren könnte [69]. Dies ist in Anbetracht der hohen medialen Präsenz und der damit verbundenen Erwartungshaltung sowohl bei Teilnehmenden als auch bei Forschenden besonders kritisch. Die tatsächliche Wirksamkeit der Substanzen könnte daher durch prononcierte Placeboeffekte überlagert sein. Dies unterstreicht die Bedeutung von Studiendesigns, welche die Effekte von Psychedelika objektiver erfassen können. Der Einsatz aktiver Placebos wie bspw. Niacin oder Methylphenidat, die Erhebung von Erwartung und Verblindungserfolg und Cross-over-Designs zur Verringerung von Noceboeffekten in Vergleichsgruppen würden hier zu einer valideren Evidenzlage beitragen [47, 73].

Auch die ethischen Herausforderungen sind nicht trivial. Psychedelika induzieren tiefgreifende Bewusstseinsveränderungen, deren Auswirkungen von Patient:innen vorab nur schwer vollständig verstanden und antizipiert werden können. Die komplexe Natur dieser Erfahrungen stellt hohe Anforderungen an die Aufklärung und Betreuung von Patient:innen, um sicherzustellen, dass diese die Tragweite und mögliche Konsequenzen ihrer Entscheidung zur Teilnahme an solchen Therapien verstehen. Des Weiteren muss das Potenzial für einen therapeutischen (Macht‑)Missbrauch und die daraus resultierende Notwendigkeit regulatorischer Maßnahmen beachtet werden. Die Vergangenheit hat gezeigt, dass die therapeutische Anwendung von Psychedelika zu unsachgemäßen Kontexten führen kann. Eine strenge Überwachung der klinischen Praxis und der Einhaltung ethischer Richtlinien ist daher unerlässlich.

## Fazit für die Praxis

- Zusammenfassend zeigen Psychedelika und Dissoziativa bisher eine hohe potenzielle Wirksamkeit und ein gutes akutes Sicherheitsprofil. Dennoch bleiben noch viele Fragen insbesondere zu den positiven wie negativen Langzeiteffekten unbeantwortet – bedeutende Forschungslücken, die angesichts der großen Aufmerksamkeit stellenweise unbeachtet bleiben.
- Dies stellt uns vor ethische, wissenschaftliche und klinische Herausforderungen, die berücksichtigt werden müssen, bevor die klinische Umsetzung in einem breiteren klinischen Kontext erfolgen kann.

## References

[1] Aday JS, Horton D, Fernandes-Osterhold G et al (2024) Psychedelic-assisted psychotherapy: where is the psychotherapy research? Psychopharmacology 10.1007/s00213-024-06620-x 38782821

[2] Alnefeesi Y, Chen-Li D, Krane E et al (2022) Real-world effectiveness of ketamine in treatment-resistant depression: A systematic review & meta-analysis. J Psychiatr Res 151:693–709 35688035 10.1016/j.jpsychires.2022.04.037

[3] Baggott MJ, Coyle JR, Erowid E et al (2011) Abnormal visual experiences in individuals with histories of hallucinogen use: a Web-based questionnaire. Drug Alcohol Depend 114:61–67 21035275 10.1016/j.drugalcdep.2010.09.006

[4] Barrett FS, Carbonaro TM, Hurwitz E et al (2018) Double-blind comparison of the two hallucinogens psilocybin and dextromethorphan: effects on cognition. Psychopharmacology 235:2915–2927 30062577 10.1007/s00213-018-4981-x PMC6162157

[5] Bell RF (2012) Ketamine for chronic noncancer pain. Curr Opin Support Palliat Care 6:183–187 22436323 10.1097/SPC.0b013e328352812c

[6] Bonnet U (2015) Long-Term Ketamine Self-Injections in Major Depressive Disorder: Focus on Tolerance in Ketamine’s Antidepressant Response and the Development of Ketamine Addiction. J Psychoactive Drugs 47:276–285 26317449 10.1080/02791072.2015.1072653

[7] Bouso JC, Palhano-Fontes F, Rodríguez-Fornells A et al (2015) Long-term use of psychedelic drugs is associated with differences in brain structure and personality in humans. Eur Neuropsychopharmacol 25:483–492 25637267 10.1016/j.euroneuro.2015.01.008

[8] Breeksema JJ, Kuin BW, Kamphuis J et al (2022) Adverse events in clinical treatments with serotonergic psychedelics and MDMA: A mixed-methods systematic review. J Psychopharmacol 36:1100–1117 36017784 10.1177/02698811221116926 PMC9548934

[9] Bremler R, Katati N, Shergill P et al (2023) Case analysis of long-term negative psychological responses to psychedelics. Sci Rep 13:10.1038/s41598-023-41145-x PMC10519946 37749109

[10] Burdick BV, Adinoff B (2013) A proposal to evaluate mechanistic efficacy of hallucinogens in addiction treatment. Am J Drug Alcohol Abuse 39:291–297 23968172 10.3109/00952990.2013.811513

[11] Butler M, Jelen L, Rucker J (2022) Expectancy in placebo-controlled trials of psychedelics: if so, so what? Psychopharmacology 239:3047–3055 36063208 10.1007/s00213-022-06221-6 PMC9481484

[12] Capela JP, Da Costa Araújo S, Costa VM et al (2013) The neurotoxicity of hallucinogenic amphetamines in primary cultures of hippocampal neurons. Neurotoxicology 34:254–263 22983118 10.1016/j.neuro.2012.09.005

[13] Capela JP, Ruscher K, Lautenschlager M et al (2006) Ecstasy-induced cell death in cortical neuronal cultures is serotonin 2A-receptor-dependent and potentiated under hyperthermia. Neuroscience 139:1069–1081 16504407 10.1016/j.neuroscience.2006.01.007

[14] Carhart-Harris RL, Kaelen M, Whalley MG et al (2014) LSD enhances suggestibility in healthy volunteers. Psychopharmacology 232:785–794 25242255 10.1007/s00213-014-3714-z

[15] Cohen S (1960) Lysergic acid diethylamide: side effects and complications. J Nerv Ment Dis 130:30–40 13811003 10.1097/00005053-196001000-00005

[16] Davis MT, Dellagiogia N, Maruff P et al (2021) Acute cognitive effects of single-dose intravenous ketamine in major depressive and posttraumatic stress disorder. Transl Psychiatry 11:10.1038/s41398-021-01327-5 PMC8032778 33833217

[17] De Greef K (2022) The Pied Piper of Psychedelic Toads. In: The New Yorker. Advance Magazine Publishers, New York

[18] Dupuis D (2021) Psychedelics as Tools for Belief Transmission. Set, Setting, Suggestibility, and Persuasion in the Ritual Use of Hallucinogens. Front Psychol 12:10.3389/fpsyg.2021.730031 PMC8651242 34887799

[19] Dupuis D, Veissière S (2022) Culture, context, and ethics in the therapeutic use of hallucinogens: Psychedelics as active super-placebos? Transcult Psychiatry 59:571–578 36263513 10.1177/13634615221131465

[20] El-Mallakh RS, Halpern JH, Abraham HD (2015) Substance Abuse: Hallucinogen- and MDMA-Related Disorders, S 1480–1506

[21] Emcdda (2023) European Drug Report 2023: Trends and Developments. EMCDDA, Lissabon

[22] Evans J, Robinson OC, Argyri EK et al (2023) Extended difficulties following the use of psychedelic drugs: A mixed methods study. PLoS ONE 18:e293349 37874826 10.1371/journal.pone.0293349 PMC10597511

[23] Fernández-Calderón F, Cleland CM, Palamar JJ (2018) Polysubstance use profiles among electronic dance music party attendees in New York City and their relation to use of new psychoactive substances. Addict Behav 78:85–93 29128711 10.1016/j.addbeh.2017.11.004 PMC5783759

[24] Food and Drug Administration (2023) Psychedelic Drugs: Considerations for Clinical Investigations Guidance for Industry. In: Food and Drug Administration, Silver Spring

[25] Gashi L, Sandberg S, Pedersen W (2021) Making “bad trips” good: How users of psychedelics narratively transform challenging trips into valuable experiences. Int J Drug Policy 87:102997 33080454 10.1016/j.drugpo.2020.102997

[26] Goodwin GM, Aaronson ST, Alvarez O et al (2022) Single-dose psilocybin for a treatment-resistant episode of major depression. N Engl J Med 387:1637–1648 36322843 10.1056/NEJMoa2206443

[27] Goodwin GM, Malievskaia E, Fonzo GA et al (2024) Must Psilocybin Always “Assist Psychotherapy”? Am J Psychiatry 181:20–25 37434509 10.1176/appi.ajp.20221043

[28] Gründer G, Brand M, Mertens LJ et al (2024) Treatment with psychedelics is psychotherapy: beyond reductionism. Lancet Psychiatry 11:231–236 38101439 10.1016/S2215-0366(23)00363-2

[29] Gukasyan N, Davis AK, Barrett FS et al (2022) Efficacy and safety of psilocybin-assisted treatment for major depressive disorder: Prospective 12-month follow-up. J Psychopharmacol 36:151–158 35166158 10.1177/02698811211073759 PMC8864328

[30] Hayes C, Wahba M, Watson S (2022) Will psilocybin lose its magic in the clinical setting? Therapeutic Advances in 12:204512532210908 10.1177/20451253221090822 PMC9036342 35480296

[31] Hendricks PS, Thorne CB, Clark CB et al (2015) Classic psychedelic use is associated with reduced psychological distress and suicidality in the United States adult population. J Psychopharmacol 29:280–288 25586402 10.1177/0269881114565653

[32] Huang M‑C, Lin S‑K (2020) Ketamine Abuse: Past and Present. In: Hashimoto K, Ide S, Ikeda K (Hrsg) Ketamine. Springer, Singapore, S 1–14

[33] Jansen KL, Darracot-Cankovic R (2001) The nonmedical use of ketamine, part two: A review of problem use and dependence. J Psychoactive Drugs 33:151–158 11476262 10.1080/02791072.2001.10400480

[34] Kious B, Schwartz Z, Lewis B (2022) Should we be leery of being Leary? Concerns about psychedelic use by psychedelic researchers. J Psychopharmacol 37:45–48 36377548 10.1177/02698811221133461

[35] Ko K, Kopra EI, Cleare AJ et al (2023) Psychedelic therapy for depressive symptoms: A systematic review and meta-analysis. J Affect Disord 322:194–204 36209780 10.1016/j.jad.2022.09.168

[36] Kramer EN, Reddy K, Shapiro B (2023) A suicide attempt following psilocybin ingestion in a patient with no prior psychiatric history. Psychiatry Res Case Reports 2:100118 10.1016/j.psycr.2023.100118

[37] Lahti A (1995) Subanesthetic Doses of Ketamine Stimulate Psychosis in Schizophrenia. Neuropsychopharmacology 13:9–19 8526975 10.1016/0893-133X(94)00131-I

[38] Leger RF, Unterwald EM (2021) Assessing the effects of methodological differences on outcomes in the use of psychedelics in the treatment of anxiety and depressive disorders: A systematic review and meta-analysis. J Psychopharmacol 36:20–30 34519567 10.1177/02698811211044688

[39] Liu Y‑L, Bavato F, Chung A‑N et al (2021) Neurofilament light chain as novel blood biomarker of disturbed neuroaxonal integrity in patients with ketamine dependence. World J Biol Psychiatry 22:713–721 33783299 10.1080/15622975.2021.1907709

[40] Liu Y, Lin D, Wu B et al (2016) Ketamine abuse potential and use disorder. Brain Res Bull 126:68–73 27261367 10.1016/j.brainresbull.2016.05.016

[41] Lodge D, Mercier MS (2015) Ketamine and phencyclidine: the good, the bad and the unexpected. Br J Pharmacol 172:4254–4276 26075331 10.1111/bph.13222 PMC4556466

[42] Malcolm B, Thomas K (2021) Serotonin toxicity of serotonergic psychedelics. Psychopharmacology 239:1881–1891 34251464 10.1007/s00213-021-05876-x

[43] Mann JJ, Michel CA, Auerbach RP (2021) Improving Suicide Prevention Through Evidence-Based Strategies: A Systematic Review. Am J Psychiatry 178:611–624 33596680 10.1176/appi.ajp.2020.20060864 PMC9092896

[44] Marks M, Brendel RW, Shachar C et al (2024) Essentials of Informed Consent to Psychedelic Medicine. JAMA Psychiatry 10.1001/jamapsychiatry.2024.0184 38598209

[45] Mcglothlin WH, Arnold DO (1971) LSD revisited: A ten-year follow-up of medical LSD use. Arch Gen Psychiatry 24:35–49 5538851 10.1001/archpsyc.1971.01750070037005

[46] Mcintyre RS, Bitter I, Buyze J et al (2024) Safety and tolerability of esketamine nasal spray versus quetiapine extended release in patients with treatment resistant depression. Eur Neuropsychopharmacol 85:58–65 38954874 10.1016/j.euroneuro.2024.05.009

[47] Mertens LJ, Koslowski M, Betzler F et al (2022) Methodological challenges in psychedelic drug trials: Efficacy and safety of psilocybin in treatment-resistant major depression (EPIsoDE) – Rationale and study design. Neurosci Appl 1:100104 10.1016/j.nsa.2022.100104 PMC12244097 40656230

[48] Metaxa A‑M, Clarke M (2024) Efficacy of psilocybin for treating symptoms of depression: systematic review and meta-analysis. BMJ e078084:10.1136/bmj-2023-078084 PMC11062320 38692686

[49] Morgan CJ, Curran HV (2012) Independent Scientific Committee on Drugs. Addiction 107:27–38 21777321 10.1111/j.1360-0443.2011.03576.x

[50] Morrison RL, Fedgchin M, Singh J et al (2018) Effect of intranasal esketamine on cognitive functioning in healthy participants: a randomized, double-blind, placebo-controlled study. Psychopharmacology 235:1107–1119 29392371 10.1007/s00213-018-4828-5 PMC5869899

[51] Müller F, Kraus E, Holze F et al (2022) Flashback phenomena after administration of LSD and psilocybin in controlled studies with healthy participants. Psychopharmacology 239:1933–1943 35076721 10.1007/s00213-022-06066-z PMC9166883

[52] Nayak S, Johnson MW (2020) Psychedelics and Psychotherapy. Pharmacopsychiatry 54:167–175 33285578 10.1055/a-1312-7297

[53] Nichols DE, Johnson MW, Nichols CD (2017) Psychedelics as Medicines: An Emerging New Paradigm. Clin Pharmacol Ther 101:209–219 28019026 10.1002/cpt.557

[54] Nikayin S, Murphy E, Krystal JH et al (2022) Long-term safety of ketamine and esketamine in treatment of depression. Expert Opin Drug Saf 21:777–787 35416105 10.1080/14740338.2022.2066651

[55] Noppers IM, Niesters M, Aarts LPHJ et al (2011) Drug-induced liver injury following a repeated course of ketamine treatment for chronic pain in CRPS type 1 patients: A report of 3 cases. Pain 152:2173–2178 21546160 10.1016/j.pain.2011.03.026

[56] Noworyta-Sokołowska K, Kamińska K, Kreiner G et al (2016) Neurotoxic Effects of 5‑MeO-DIPT: A Psychoactive Tryptamine Derivative in Rats. Neurotox Res 30:606–619 27461536 10.1007/s12640-016-9654-0 PMC5047954

[57] Nutt D, King LA, Saulsbury W et al (2007) Development of a rational scale to assess the harm of drugs of potential misuse. Lancet 369:1047–1053 17382831 10.1016/S0140-6736(07)60464-4

[58] Perez N, Langlest F, Mallet L et al (2023) Psilocybin-assisted therapy for depression: A systematic review and dose-response meta-analysis of human studies. Eur Neuropsychopharmacol 76:61–76 37557019 10.1016/j.euroneuro.2023.07.011

[59] Perry EB, Cramer JA, Cho H‑S et al (2007) Psychiatric safety of ketamine in psychopharmacology research. Psychopharmacology 192:253–260 17458544 10.1007/s00213-007-0706-2

[60] Quednow BB, Kometer M, Geyer MA et al (2011) Psilocybin-Induced Deficits in Automatic and Controlled Inhibition are Attenuated by Ketanserin in Healthy Human Volunteers. Neuropsychopharmacology 37:630–640 21956447 10.1038/npp.2011.228 PMC3260978

[61] Reardon S (2024) MDMA therapy for PTSD rejected by FDA panel. Nature. 10.1038/d41586-024-01622-3 38844808 10.1038/d41586-024-01622-3

[62] Rudin D, Liechti ME, Luethi D (2021) Molecular and clinical aspects of potential neurotoxicity induced by new psychoactive stimulants and psychedelics. Exp Neurol 343:113778 34090893 10.1016/j.expneurol.2021.113778

[63] Sayalı C, Barrett FS (2023) The costs and benefits of psychedelics on cognition and mood. Neuron 111:614–630 36681076 10.1016/j.neuron.2022.12.031

[64] Schlag AK, Aday J, Salam I et al (2022) Adverse effects of psychedelics: From anecdotes and misinformation to systematic science. J Psychopharmacol 36:258–272 35107059 10.1177/02698811211069100 PMC8905125

[65] Shalit N, Rehm J, Lev-Ran S (2019) Epidemiology of hallucinogen use in the U.S. results from the National epidemiologic survey on alcohol and related conditions III. Addict Behav 89:35–43 30245407 10.1016/j.addbeh.2018.09.020

[66] Short B, Fong J, Galvez V et al (2018) Side-effects associated with ketamine use in depression: a systematic review. Lancet Psychiatry 5:65–78 28757132 10.1016/S2215-0366(17)30272-9

[67] Simonsson O, Carlbring P, Carhart-Harris R et al (2023) Assessing the risk of symptom worsening in psilocybin-assisted therapy for depression: A systematic review and individual participant data meta-analysis. Psychiatry Res 327:115349 37523886 10.1016/j.psychres.2023.115349 PMC10528683

[68] Smith-Apeldoorn SY, Veraart JKE, Spijker J et al (2022) Maintenance ketamine treatment for depression: a systematic review of efficacy, safety, and tolerability. Lancet Psychiatry 9:907–921 36244360 10.1016/S2215-0366(22)00317-0

[69] Soliman PS, Curley DE, Capone C et al (2024) In the new era of psychedelic assisted therapy: A systematic review of study methodology in randomized controlled trials. Psychopharmacology 241:1101–1110 38683460 10.1007/s00213-024-06598-6 PMC11529604

[70] Swainson J, Klassen LJ, Brennan S et al (2022) Non-parenteral Ketamine for Depression: A Practical Discussion on Addiction Potential and Recommendations for Judicious Prescribing. Cns Drugs 36:239–251 35165841 10.1007/s40263-022-00897-2 PMC8853036

[71] Sznitman SR, Taubman DS (2016) Drug Use Normalization: A Systematic and Critical Mixed-Methods Review. J Stud Alcohol Drugs 77:700–709 27588528 10.15288/jsad.2016.77.700

[72] Timmermann C, Kettner H, Letheby C et al (2021) Psychedelics alter metaphysical beliefs. Sci Rep 11:22166 34815421 10.1038/s41598-021-01209-2 PMC8611059

[73] Van Elk M, Fried EI (2023) History repeating: guidelines to address common problems in psychedelic science. Therapeutic Advances in 13:10.1177/20451253231198466 PMC10521293 37766730

[74] Vargas MV, Dunlap LE, Dong C et al (2023) Psychedelics promote neuroplasticity through the activation of intracellular 5‑HT2A receptors. Science 379:700–706 36795823 10.1126/science.adf0435 PMC10108900

[75] Vines L, Sotelo D, Johnson A et al (2022) Ketamine use disorder: preclinical, clinical, and neuroimaging evidence to support proposed mechanisms of actions. Intell Med 2:61–68 35783539 10.1016/j.imed.2022.03.001 PMC9249268

[76] Von Rotz R, Schindowski EM, Jungwirth J et al (2023) Single-dose psilocybin-assisted therapy in major depressive disorder: a placebo-controlled, double-blind, randomised clinical trial. eClinicalMedicine 56:101809 36636296 10.1016/j.eclinm.2022.101809 PMC9830149

[77] Xu K, Lipsky RH (2014) Repeated ketamine administration alters N‑methyl-d-aspartic acid receptor subunit gene expression: Implication of genetic vulnerability for ketamine abuse and ketamine psychosis in humans. Exp Biol Med (Maywood) 240:145–155 25245072 10.1177/1535370214549531 PMC4469194

[78] Yao Y, Guo D, Lu T‑S et al (2024) Efficacy and safety of psychedelics for the treatment of mental disorders: A systematic review and meta-analysis. Psychiatry Res 335:115886 38574699 10.1016/j.psychres.2024.115886

[79] Yerubandi A, Thomas JE, Bhuiya NMMA et al (2024) Acute Adverse Effects of Therapeutic Doses of Psilocybin: A Systematic Review and Meta-Analysis. Jama Netw Open 7:e245960–e245960 38598236 10.1001/jamanetworkopen.2024.5960 PMC11007582

[80] Zeifman RJ, Singhal N, Breslow L et al (2021) On the relationship between classic psychedelics and suicidality: a systematic review. Acs Pharmacol Transl Sci 4:436–451 33860173 10.1021/acsptsci.1c00024 PMC8033757

