# Erratum zu: Psychedelika und Dissoziativa in der Psychiatrie: Herausforderungen in der Behandlung

**DOI:** 10.1007/s00115-024-01744-z

**Published:** 2024-09-04

**Authors:** Johannes Jungwirth, Francesco Bavato, Boris B. Quednow

**Affiliations:** 1 AG Neurophänomenologie des Bewusstseins, Erwachsenenpsychiatrie und Psychotherapie, Psychiatrische Universitätsklinik Zürich, Universität Zürich, Zürich, Schweiz; 2 AG Experimentelle Pharmakopsychologie und Psychologische Suchtforschung, Erwachsenenpsychiatrie und Psychotherapie, Psychiatrische Universitätsklinik Zürich, Universität Zürich, Zürich, Schweiz

**Erratum zu:**

**Nervenarzt 2024**

10.1007/s00115-024-01727-0

In diesem Artikel waren die Daten zur institutionellen Zugehörigkeit für Johannes Jungwirth falsch angegeben.

Statt „AG Experimentelle Pharmakopsychologie und psychologische Suchtforschung, Erwachsenenpsychiatrie und Psychotherapie, Psychiatrische Universitätsklinik Zürich, Universität Zürich, Zürich, Schweiz“ hätte sie „AG Neurophänomenologie des Bewusstseins, Erwachsenenpsychiatrie und Psychotherapie, Psychiatrische Universitätsklinik Zürich, Universität Zürich, Zürich, Schweiz“ lauten sollen.

Der Originalbeitrag wurde korrigiert.

